# A likely unavoidable clinical scenario during treatment for venous thromboembolism complicated with severe immune thrombocytopenia: A case report

**DOI:** 10.1002/ccr3.4805

**Published:** 2021-09-18

**Authors:** Takaya Takeuchi, Yunosuke Matsuura, Yoshimasa Yamamura, Momoko Ohkusu, Shohei Koyama, Takeshi Kawaguchi, Mayumi Akaki Nagayasu, Hiroyuki Komatsu, Akihiko Okayama, Tetsunori Ishikawa, Tatsuya Atsumi, Kazuo Kitamura

**Affiliations:** ^1^ Department of Internal Medicine, Circulatory and Body Fluid Regulation University of Miyazaki Miyazaki Japan; ^2^ Center for Post‐Graduation Clinical Training Faculty of Medicine University of Miyazaki Miyazaki Japan; ^3^ Department of Rheumatology Infectious Diseases and Laboratory Medicine University of Miyazaki Miyazaki Japan; ^4^ Clinical Laboratory University of Miyazaki Hospital Miyazaki Japan; ^5^ Section of Oncopathology and Regenerative Biology Department of Pathology Faculty of Medicine University of Miyazaki Miyazaki Japan; ^6^ Department of Rheumatology, Endocrinology and Nephrology Faculty of Medicine and Graduate School of Medicine Hokkaido University Sapporo Japan

**Keywords:** anti‐thrombotic therapy, immune thrombocytopenia, venous thromboembolism

## Abstract

Patients with immune thrombocytopenia have increased risks of bleeding and thrombosis. The acute‐phase treatment for venous thromboembolism complicated with severe immune thrombocytopenia involves a “platelet dilemma” in therapeutic decision‐making.

## Graphical abstract

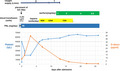

## 1 INTRODUCTION

Immune thrombocytopenia is associated with increased risks of bleeding and thrombosis. The treatment for venous thromboembolism complicated with severe thrombocytopenia involves a “platelet dilemma” in therapeutic decision‐making. Thrombotic exacerbation should be considered when increasing the platelet count to initiate anti‐coagulation therapy in cases that involve immune thrombocytopenia.

Immune thrombocytopenia (ITP) is an autoimmune disease characterized by an increased risk of bleeding. Furthermore, recent reports have described a paradoxically increased incidence of arterial and venous thromboembolic events in patients with ITP compared with that in patients without ITP.^1^ Thus, a lower platelet count in cases of ITP does not prevent thrombotic complications.

The co‐existence of thrombotic and bleeding risks, which is frequently observed in cases of cancer‐associated thrombosis, creates a therapeutic dilemma regarding the trade‐offs that are needed to manage their risks. Patients with ITP are similarly exposed to both risks, although the underlying mechanism is different from that in cancer cases. It would be useful to establish therapeutic guidelines for managing ITP‐associated thrombosis; however, guideline development is complicated by the complex and heterogeneous clinical background of ITP‐associated thrombosis.^2^ Therefore, the accumulation of longitudinal case information is needed to better understand the clinical scenarios wherein thrombotic complications are encountered in ITP cases. We report a case involving severe ITP with venous thromboembolism (VTE) and discuss the clinical dilemma regarding managing the risks of ITP‐associated thrombosis and its related treatments.

## 2 CASE REPORT

A 51‐year‐old woman was referred to our hospital because of sudden‐onset right leg edema and dyspnea. At admission, her blood pressure was 120/70 mmHg, heart rate was 70/min, and oxygen saturation was 98% (2 L/min oxygen supply via a nasal cannula). Despite the vital signs implying a relatively stable hemodynamic profile, we observed bleeding signs that included purpura in the lower extremities, epistaxis, and petechiae of the hard palate. The patient's medical records revealed a history of smoking and diagnosis of ITP, with splenectomy performed when she was a teenager. Following over 20‐year disease‐free period after splenectomy, the patient experienced ITP relapse and was subsequently diagnosed with mixed connective tissue disease (MCTD). The patient had subsequently been treated for secondary ITP using 20 mg/day of oral prednisolone (PSL).

**FIGURE 1:**
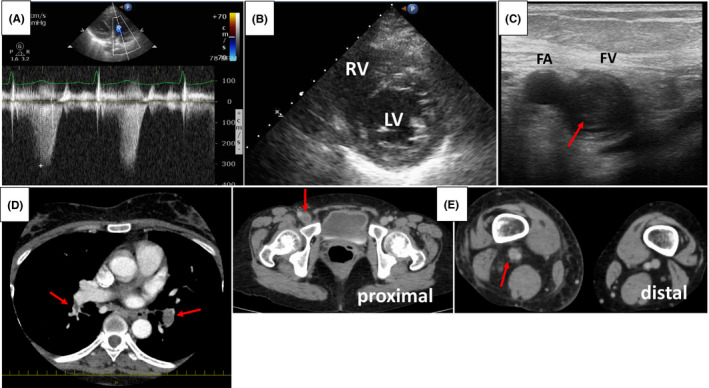
Findings from echocardiography, lower extremity ultrasonography, and computed tomography regarding venous thromboembolism. Echocardiography shows a mildly increased tricuspid regurgitation peak gradient (A) and no evidence of a D‐shaped left ventricle in the short axis view (B). RV: right ventricle. (C): Lower extremity ultrasonography reveals a partially floating thrombus (red arrow) with low‐to‐isometric echogenicity in the proximal femoral vein. FA: femoral artery, FV: femoral vein. (D, E): Contrast‐enhanced computed tomography reveals bilateral pulmonary artery embolic thrombi (red arrows, D) and deep vein thrombosis extending from proximal to distal in the right femoral vein

Echocardiography performed at admission revealed mild pulmonary hypertension (tricuspid regurgitation pressure gradient: 39 mmHg, Figure 1A) without a D‐shaped left ventricle (Figure 1B). In addition, an ultrasonography (US) examination for deep vein thrombosis (DVT) revealed low‐echoic and partially floating thrombi (Figure 1C) in the proximal femoral vein. Contrast‐enhanced computed tomography (CT) revealed obvious thromboembolism in the bilateral pulmonary arteries (Figure 1D) and thrombi extending from proximal to distal in the femoral vein (Figure 1E). Based on these signs, the patient was diagnosed with hemodynamically stable pulmonary thromboembolism (PTE) and fresh proximal DVT, which required anti‐coagulation treatment.

**FIGURE 2:**
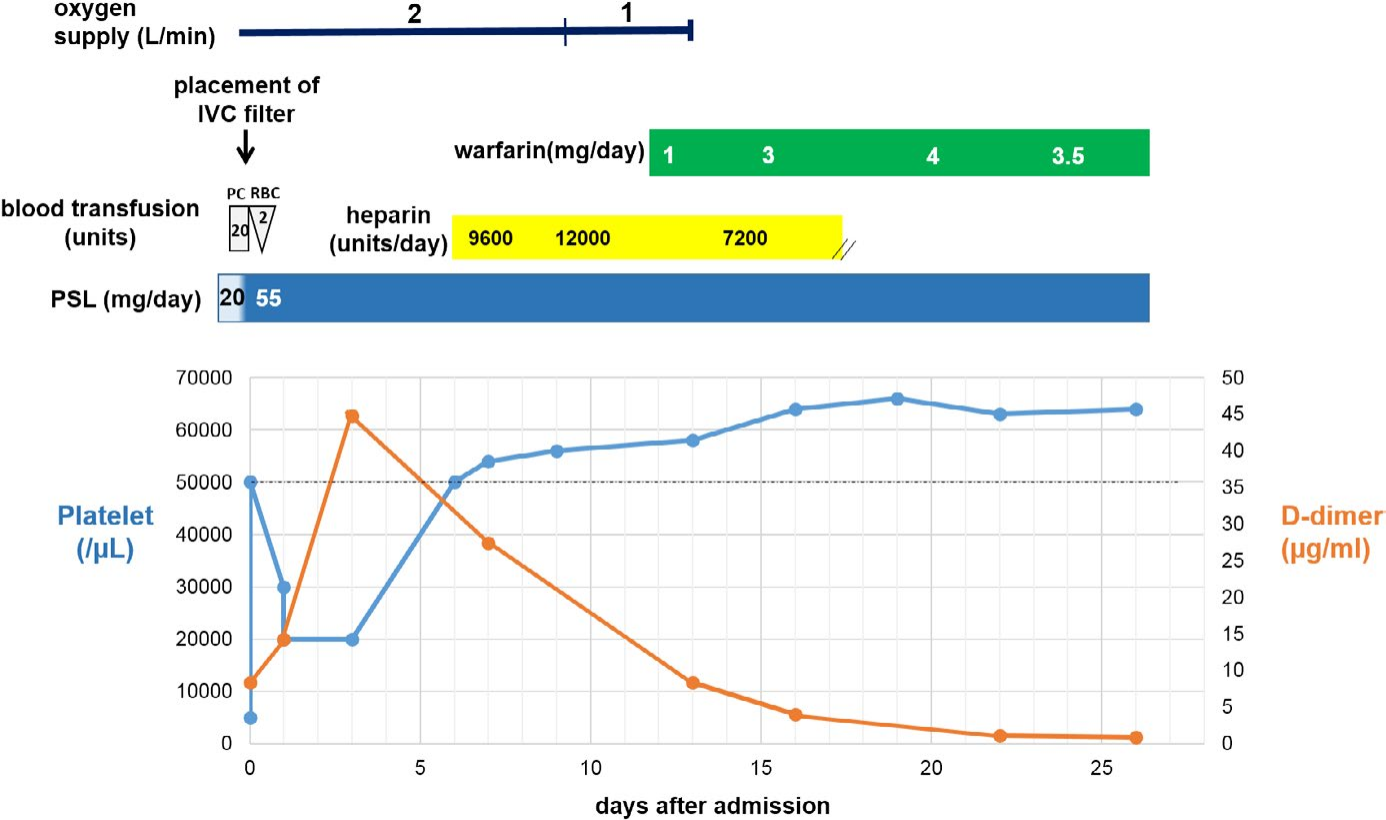
Therapeutic regimen and changes in platelet count and D‐dimer concentrations during treatment for acute venous thromboembolism until a negative D‐dimer result was obtained (<1.0 µg/ml). The dotted line indicates a platelet count of 50,000/µL as a threshold for tolerable anti‐coagulation. PC: platelet, RBC: red blood cells, PSL: prednisolone, and IVC: inferior vena cava

At admission, the blood test revealed highly severe thrombocytopenia (5 × 10^3^/μl), which suggested that immediate initiation of anti‐coagulation treatment for VTE was inadvisable based on the risk of fatal bleeding. Thus, a temporary inferior vena cava (IVC) filter was placed after a platelet transfusion. In addition, the PSL dose was increased to 1 mg/kg to avoid bleeding. Figure 2 shows the therapeutic regimen and changes in platelet counts and D‐dimer concentrations during the treatment for acute VTE until a negative D‐dimer result was observed (<1.0 µg/ml).

The platelet counts responded to the increased PSL dose; however, the right lower extremity edema worsened (vs. at admission), and blood tests revealed a dramatically increased D‐dimer concentration (44 µg/ml). Follow‐up using US and CT confirmed that DVT apparently worsened in the right lower extremity; however, thrombi in the pulmonary arteries remained unchanged, and no evidence of thrombus‐induced filter occlusion was observed. The platelet count reached 5 × 10^4^/μl on day 7, and anti‐coagulation treatment was initiated using unfractionated heparin, which was subsequently changed to warfarin therapy to achieve a prothrombin time/international normalized ratio of 2.0. The D‐dimer concentrations rapidly decreased in response to the anti‐coagulation treatment, and we confirmed a negative D‐dimer result on day 27. On day 35, the CT images revealed no large thrombi in the deep leg veins and pulmonary arteries, and the IVC filter was finally removed on day 42. The patient did not experience any worsening of bleeding signs during the clinical course.

During the treatment course, we also investigated the underlying risk of thrombosis. Table 1 shows the results of blood tests, which included factors and autoantibodies associated with coagulation disorders, connective tissue diseases, and anti‐phospholipid syndrome. The clinical manifestations and blood test results at admission indicated that the concomitant autoimmune disease was systemic lupus erythematosus (SLE) rather than MCTD. Although anemia subsequent to the thrombocytopenia was also present, no evidence of hemolysis was observed. In addition, anti‐cardiolipin antibodies were detected at a low titer; however, we did not detect anti‐cardiolipin‐β2‐glycoprotein I antibodies, lupus anticoagulants, or a prolonged activated partial thromboplastin time. Therefore, the laboratory data and clinical manifestations, including pregnancy history, did not satisfy the diagnostic criteria for anti‐phospholipid syndrome. In addition, weakly positive results were observed for the immunoglobulin (IG) M subtype of anti‐phosphatidylserine/prothrombin antibodies. Bone marrow aspiration revealed normal to mildly hypercellular marrow and an increase in mature megakaryocyte count, which did not contradict the diagnosis of ITP or provide any evidence of malignancy.

**TABLE 1:** Laboratory data at admission

Peripheral blood		Biochemistry		Immunological test	
WBC (×10³/µl)	8.3 (3.3–8.6)	TP (g/dl)	6.84 (6.6–8.1)	ANA (fold)	1280 (<40)
Neutrophil (%)	85.4 (37–72)	Albumin (g/dl)	2.36 (4.1–5.1)	C3 (mg/dl)	58 (73–138)
Lymphocyte (%)	13.6 (20–50)	T‐bil (mg/dl)	0.3 (0.4–1.5)	C4 (mg/dl)	4 (11–31)
Monocyte (%)	0.8 (4.1–10.6)	D‐bil (mg/dl)	0.1 (0.05–0.3)	CH 50 (/ml)	14 (28–55)
Eosinophil (%)	0 (0.6–8.3)	AST (U/L)	28 (13–30)	Anti‐SSA ab (U/ml)	155 (<10)
Basophil (%)	0.2 (0–1.3)	ALT (U/L)	14 (7–23)	Anti‐SSB ab (U/ml)	<1 (<10)
RBC (×10⁶/µl)	3.14 (3.86–4.92)	LD (U/L)	289 (124–222)	Anti‐Sm ab (U/ml)	2 (<7)
Hemoglobin (g/dl)	8.4 (11.6–14.8)	CK (U/L)	69 (41–153)	Anti‐dsDNA IgG ab (IU/ml)	19 (<12)
Hematocrit (%)	26.4 (35.1–44.4)	BUN (mg/dl)	13.4 (8–20)	Anti‐RNP ab (U/ml)	2.1 (<10)
MCV (fL)	84.1 (83.6–98.2)	Cre (mg/dl)	0.56 (0.46–0.79)	Anti‐CLβ2GPI ab	<1.2 (<3.5)
MCHC (g/dl)	31.8 (31.7–35.3)	Na (mmol/L)	136 (138–145)	Anti‐PS‐PT IgM (U)	42.9 (≦30)
Reticulocyte (%)	3.1 (0.8–2.2)	K (mmol/L)	3.6 (3.6–4.8)	Anti‐PS‐PT IgG (U)	18.6 (≦30)
Platelet (×10³/µl)	5 (158–348)	Cl (mmol/L)	107 (101–108)	Lupus Anticoagulant	1.17 (<1.3)
		BNP (pg/ml)	87.7 (<18.4)	(Before/after, sec)	42.7/36.4
**Coagulation**		CRP (mg/dl)	1.07 (0–0.14)	PA‐IgG (ng/10⁷cells)	1760 (<46)
PT (sec)	12.1 (10.5–12.5)	Ferritin (ng/ml)	16 (12–60)	Anti‐cardiolipin IgM ab	15 (<8)
PT‐INR	1.12	UIBC (µg/dl)	261 (137–325)	Anti‐cardiolipin IgG ab	20 (<10)
APTT (sec)	29.6 (25–35)	TIBC (µg/dl)	279 (236–406)	Helicobacter pylori ab	<3 (<10)
FDP (µg/ml)	25.6 (<5)	Fe (µg/dl)	18 (40–188)		
AT‐III (%)	88 (80–130)	**Arterial blood gas analysis (2 L/min O2 supply)**			
Fibrinogen (mg/dl)	207 (200–400)	pH	7.49 (7.35–7.45)		
D‐dimer (µg/ml)	8.31 (0–1)	pCO2 (mmHg)	31.3 (27–39)		
Protein S activity (%)	40 (60–150)	pO2 (mmHg)	100 (83–108)		
Protein C activity (%)	75 (54–146)	HCO3 (mmol/L)	23.6 (21.2–28.3)		
ADAMTS13 activity (%)	52	BE (mmol/L)	0.9 (−2–3)		

Abbreviations: ADAMTS13, A Disintegrin‐like and Metalloproteinase with Thrombospondin type 1 motifs 13; ALT, Alanine aminotransferase; ANA, Antinuclear Antibody; APTT, Activated Partial Thromboplastin Time; AST, Aspartate aminotransferase; AT‐III, Antithrombin‐III; T‐bil, Total bilirubin; BE, Base Excess; BNP, Brain natriuretic peptide ab antibody; BUN, Blood urea nitrogen; CK, Creatinine Kinase; CLβ2GPI, Cardiolipin‐β2‐Glycoprotein I; Cre, Creatinine; CRP C‐reactive protein; D‐bil, Direct bilirubin; dsDNA, double‐stranded Deoxyribonucleic Acid; FDP, Fibrinogen Degradation Products; LD, Lactate Dehydrogenase; MCHC, Mean Corpuscular Hemoglobin Concentration; MCV, Mean Corpuscular Volume; PA‐IgG, Platelet‐Associated Immunoglobulin G; PS‐PT, Phosphatidylserine Prothrombin; PT, Prothrombin Time; PT‐INR Prothrombin Time‐International Normalized Ratio; RBC, Red Blood Cell; RNP, Ribonucleoprotein; TIBC, Total iron binding capacity; TP, Total protein; UIBC, Unsaturated iron binding capacity; WBC, White Blood Cell.

## 3 DISCUSSION

Our patient developed VTE even with severe thrombocytopenia and bleeding signs. Although this pathological scenario is seemingly paradoxical, multiple recent studies have demonstrated that patients with ITP have an increased risk of thrombotic complications compared with those without ITP.^1^, ^3^ In addition, ITP‐related risk factors for developing thrombotic complications have been reported.^4^ Similarly, our case involved various risk factors for ITP‐related thrombosis, including steroid use,^5^, ^6^ a history of smoking,^7^ positive anti‐phospholipid antibodies,^8^, ^9^, ^10^ comorbid SLE,^1^ and a history of splenectomy.^11^, ^12^, ^13^ However, even in ITP cases involving these risk factors, the magnitude of thrombotic risk that is needed to justify the initiation of preventative anti‐thrombotic therapy remains unclear, especially given the trade‐off regarding the increased risk of bleeding.^14^ Balitsky et al. proposed a simple risk assessment score that might be helpful for balancing the thrombotic and bleeding risks during anti‐thrombotic therapy in ITP cases.^15^ In this case, this score was initially negative, indicating a trend toward a high bleeding risk even when developing VTE. However, further studies are required to develop more personalized strategies for patients with ITP.

The present case involved an intriguing clinical course in terms of immunological comorbidity. Prior medical records before admission to our hospital showed that the initial diagnosis was primary ITP (previously described as idiopathic thrombocytopenia); however, SLE was confirmed as a comorbidity at this admission. A recent population‐based study revealed that patients with primary ITP have an approximately 27‐fold increased risk of developing SLE,^16^ and another previous report revealed that 12% of patients with SLE were initially diagnosed with primary ITP.^17^ Thus, careful monitoring is needed to identify comorbid SLE, even after a diagnosis of primary ITP, as therapeutic options for SLE might be effective in cases with intractable ITP, and these treatments might make it possible to avoid splenectomy. Furthermore, newly emerged comorbidities after the diagnosis of ITP may alter the risk stratification of thrombotic complications. Therefore, using only a single diagnosis or risk stratification point may complicate the risk estimation for developing ITP‐associated thrombotic complications, and follow‐up assessments may be needed for effective thrombotic risk modeling.

There are no guidelines for managing thrombotic complications in patients with ITP, which may be related to their heterogeneous clinical background. Some experts recommend a platelet cutoff of ≥50,000/µl to start anti‐coagulation treatment for thrombotic complications,^14^, ^18^ and we used this cutoff value to guide the initiation of anti‐coagulation therapy. The currently available options for increasing platelet count include steroid treatment, IG therapy, and thrombopoietin receptor agonist treatment. In the present case, steroid treatment and IG therapy were options for increasing the platelet count; however, we selected PSL administration at an increased dose because the patient had a relatively stable hemodynamic profile, and PTE recurrence could at least be partially prevented using the IVC filter. Another option is IG therapy, which takes 1–2 days to increase platelet count.^19^ Thus, IG therapy might be preferable over steroid treatment in patients with VTE showing signs of hemodynamic collapse, given the unavoidable use of anti‐coagulation treatment during acute‐phase intensive treatment that includes circulatory support or surgery. Nevertheless, a rapid short‐term increase in the platelet count might also be associated with adverse effects, including fatal thrombotic complications.^20^ Thus, thrombogenic responses could reasonably be expected during treatment to increase platelet count using IG therapy^21^ or steroid treatment.^22^ Additionally, our patient exhibited thrombotic exacerbation in the right lower extremity with a markedly elevated D‐dimer concentration during the period when the platelet counts responded to the increased PSL dose. Although there is no clearly appropriate strategy for managing this type of thrombotic exacerbation, an IVC filter might be useful to avoid fatal PTE. The acute‐phase management of thrombotic complications in severe thrombocytopenia is extremely challenging because of this therapeutic dilemma. However, we hope that this report will provide information regarding how to manage the trade‐off between increasing platelet counts and avoiding sudden fatal thrombotic exacerbation. In addition, we are presently in the era of artificial intelligence (AI), wherein longitudinal complex clinical information could be integrated. In a case of ITP associated with thrombotic complications, if we precisely accumulate the clinical data in combination with AI use, it will be possible to establish a more personalized risk scoring system to safely manage cases of dilemma.

In conclusion, the acute‐phase treatment for VTE complicated by severe thrombocytopenia involves a “platelet dilemma” in terms of therapeutic decision‐making. Thrombotic exacerbation is an ITP‐specific clinical scenario that should be considered when increasing platelet counts to initiate anti‐coagulation therapy.

## CONFLICTS OF INTEREST

None declared.

## AUTHOR CONTRIBUTIONS

TT, MO, and YM collected the data and drafted the manuscript. TT, MO, YY, YM, SK, TI, and TK managed the patient. MAN performed the histological examination. HK, AO, TI, TA, and KK supervised the manuscript.

## ETHICAL APPROVAL

An ethical review is not required for this type of article. Written informed consent for the publication of this article was obtained from the patient.

## CONSENT

Published with written consent of the patient.

## Data Availability

The data that support the findings of this study are available from the corresponding author upon the reasonable request.
